# Oxidative Stress Modulation by Carnosine in Scaffold Free Human Dermis Spheroids Model: A Proteomic Study

**DOI:** 10.3390/ijms23031468

**Published:** 2022-01-27

**Authors:** Gilda Aiello, Francesca Rescigno, Marisa Meloni, Giovanna Baron, Giancarlo Aldini, Marina Carini, Alfonsina D’Amato

**Affiliations:** 1 Department of Human Science and Quality of Life Promotion, Telematic University San Raffaele, 00166 Rome, Italy; gilda.aiello@uniroma5.it; 2 Department of Pharmaceutical Sciences, University of Milan, 20133 Milan, Italy; giovanna.baron@unimi.it (G.B.); giancarlo.aldini@unimi.it (G.A.); marina.carini@unimi.it (M.C.); 3 VitroScreen, In Vitro Innovation Center, 20149 Milan, Italy; francesca.rescigno@vitroscreen.com (F.R.); marisa.meloni@vitroscreen.com (M.M.)

**Keywords:** carnosine, oxidative stress, dermis spheroids, high-resolution mass spectrometry, network analyses

## Abstract

Carnosine is an endogenous β-alanyl-L-histidine dipeptide endowed with antioxidant and carbonyl scavenger properties, which is able to significantly prevent the visible signs of aging and photoaging. To investigate the mechanism of action of carnosine on human skin proteome, a 3D scaffold-free spheroid model of primary dermal fibroblasts from a 50-year-old donor was adopted in combination with quantitative proteomics for the first time. The label free proteomics approach based on high-resolution mass spectrometry, integrated with network analyses, provided a highly sensitive and selective method to describe the human dermis spheroid model during long-term culture and upon carnosine treatment. Overall, 2171 quantified proteins allowed the in-depth characterization of the 3D dermis phenotype during growth and differentiation, at 14 versus 7 days of culture. A total of 485 proteins were differentially regulated by carnosine at 7 days, an intermediate time of culture. Of the several modulated pathways, most are involved in mitochondrial functionality, such as oxidative phosphorylation, TCA cycle, extracellular matrix reorganization and apoptosis. In long-term culture, functional modules related to oxidative stress were upregulated, inducing the aging process of dermis spheroids, while carnosine treatment prevented this by the downregulation of the same functional modules. The application of quantitative proteomics, coupled to advanced and relevant in vitro scaffold free spheroids, represents a new concrete application for personalized therapies and a novel care approach.

## 1. Introduction

L-carnosine is an effective molecule with antioxidant and carbonyl scavenger properties, able to influence cellular lifespan and the onset of age-related change with an apparent rejuvenation of senescent fibroblasts at physiological concentrations [1,2]. The delivery of carnosine through the stratum corneum in the presence of magnesium ions results in skin-soothing and antioxidant properties allowing protection against oxidative stress. [3]. Moreover, carnosine prevents the fibroblast senescence process caused by HNE (4-hydroxy-2-nonenal) or acrolein adducts with elastin isoforms [4,5]. Carnosine also protects skin from photodamage and the aging process induced by UVA [6]. Its mechanisms of action are still not well characterized even though several studies have demonstrated the involvement of metal ion chelation, the scavenging of reactive oxygen species (ROS) and peroxyl radicals [7]. To investigate the effect of carnosine in human skin, an appropriate and representative model of dermis should be adopted. In the present work, for the first time a novel model of dermal spheroids of primary human fibroblasts and advanced quantitative proteomics were employed in order to (i) fully characterize the dermis spheroids phenotype during long-term culture and (ii) to evaluate the protein pathways modulated by carnosine in dermis spheroids and on the components of the extracellular matrix (ECM). In particular, the ECM represents a functional compartment composed of a complex network of hundreds of proteins, such as collagens and elastin, forming a stable structure to support the surrounding cells. Besides, the ECM also interacts with cells affecting their function, growth, differentiation, migration and tissue homeostasis maintenance. Among in vitro models, spheroid tissue systems are advanced 3D in vitro models able to perfectly mimic native ECM architecture and the functionality of in vivo human tissues. Indeed, the 3D scaffold-free spheroids represent a further novel physiological tissue model, characterized by a proper 3D microenvironment built by the cells themselves during the culture time. Thanks to the self-assembly approach and to the geometrical guidance, cells grow and aggregate according to their native behavior, which could be lost in conventional 2-dimensional flat cultures [8,9]. Indeed, in the 3D microenvironment, the spatial guidance allows cells to be well organized in a 3D miniaturized micro-physiological system by preserving cellular phenotype and improving the natural functionality of native tissue. Protein network analyses, based on quantitative high-resolution mass spectrometric data, allows a more complete description of a complex matrix profile and its modulation by molecules in terms of composition and assembly. The application of this powerful approach to a new dermis spheroid model represents a useful and concrete tool for pre-clinical investigations and the application of a personalized care approach.

## 2. Results and Discussion

### 2.1. Protein Profiling of Human Dermis Spheroids Model

**Figure 1:**
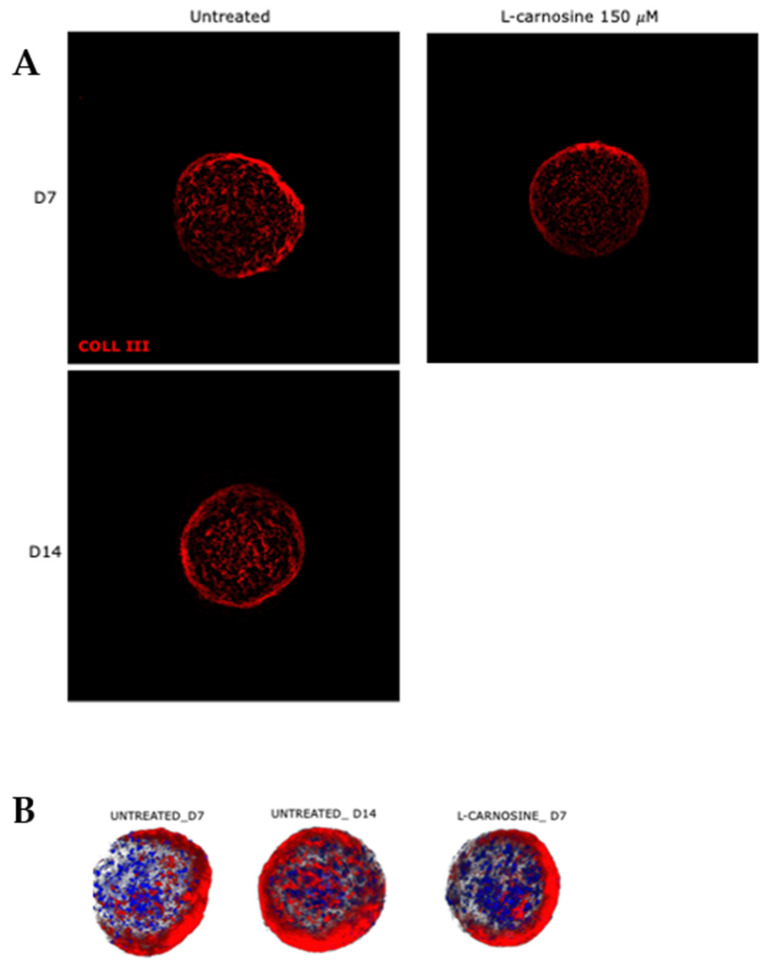
(**A**) Immunofluorescence for collagen type III (in red) on 3D whole mount tissues. Mag. 20×. (**B**) The 3D rendering of Z stack maximum projection: nuclei (stained in blue) and collagen type III signal (stained in red).

The phenotype of long-term cultured human scaffold-free dermis spheroids [9] was studied by a correlation of the proteome after 7 and 14 days of culture. To evaluate protein localization and modification during the culture time, the expression of collagen type III—an early secreted fibrillar protein during de-novo ECM deposition of fibroblasts—was analyzed by immunofluorescence. High-resolution Z-stack acquisition showed the collagen III distribution inside dermal ECM (Figure 1A). No differences of collagen III expression resulted in dermal spheroids at 7 versus 14 days of culture (Figure 1A). This indicates 3D fibroblast preservation of native phenotype and physiological establishment of a compact and dense de novo ECM during the long-term culture. Moreover, the treatment with carnosine did not show an effect on collagen type III synthesis either (Figure 1A). Quantification of signal expression confirmed this evidence (data not shown). The 3D rendering of Z-stack maximum projection revealed a well cellularized tissue with a complex structural organization, where cells (nuclei in blue) were perfectly embedded in the endogenous ECM rich in collagen fibers (Figure 1B).

**Figure 2:**
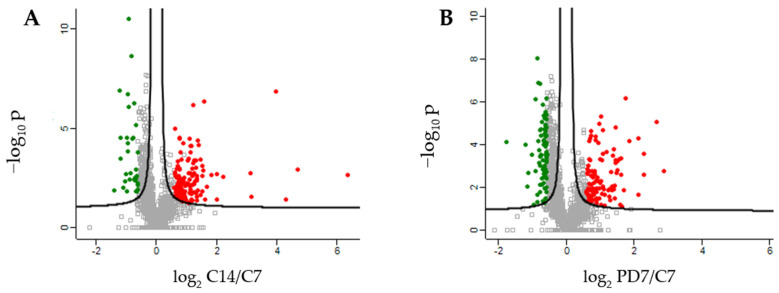
Scatter plots of log2 ratio on x-axis against -log10 *p* value on y-axis of significantly quantified proteins. (**A**) Quantified proteins in human dermis spheroids over time, from 7 to 14 days (C14 vs. C7). (**B**) Quantified proteins in carnosine pre-treated dermis versus control at 7 days (PD7 vs. C7). Green color indicates downregulation (log2 ratio ≤ −0.55), red color represents upregulation (log2 ratio ≥ 0.55). Statistical parameters (*p* < 0.05; q < 0.05, q = FDR adjusted *+* value) were set to identify the differentially expressed proteins between samples.

**Figure 3:**
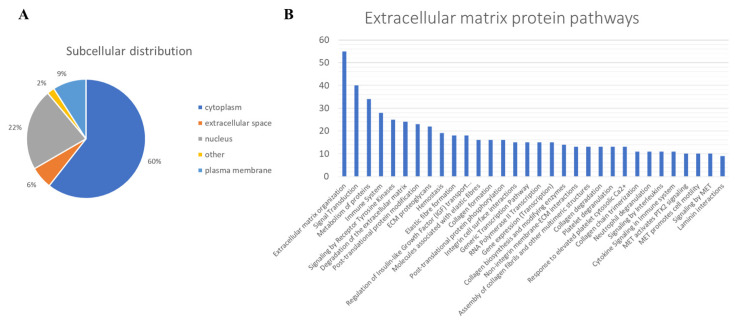
(**A**) Subcellular distribution of identified proteins. (**B**) Main pathways involving extracellular matrix organization and plasma membrane proteins.

**Figure 4:**
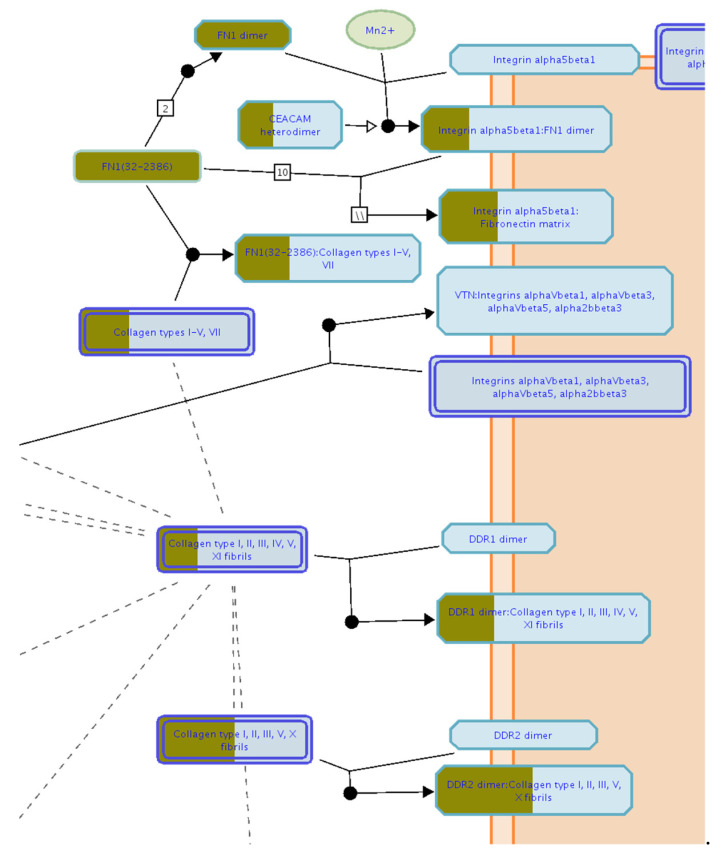
Functional module of matrix reorganization obtained by Reactome (a section of the entire picture) (Supplementary Figure S2). The brown color indicates all the extracellular and plasma proteins identified in analyses of controls and carnosine treated samples.

**Figure 5:**
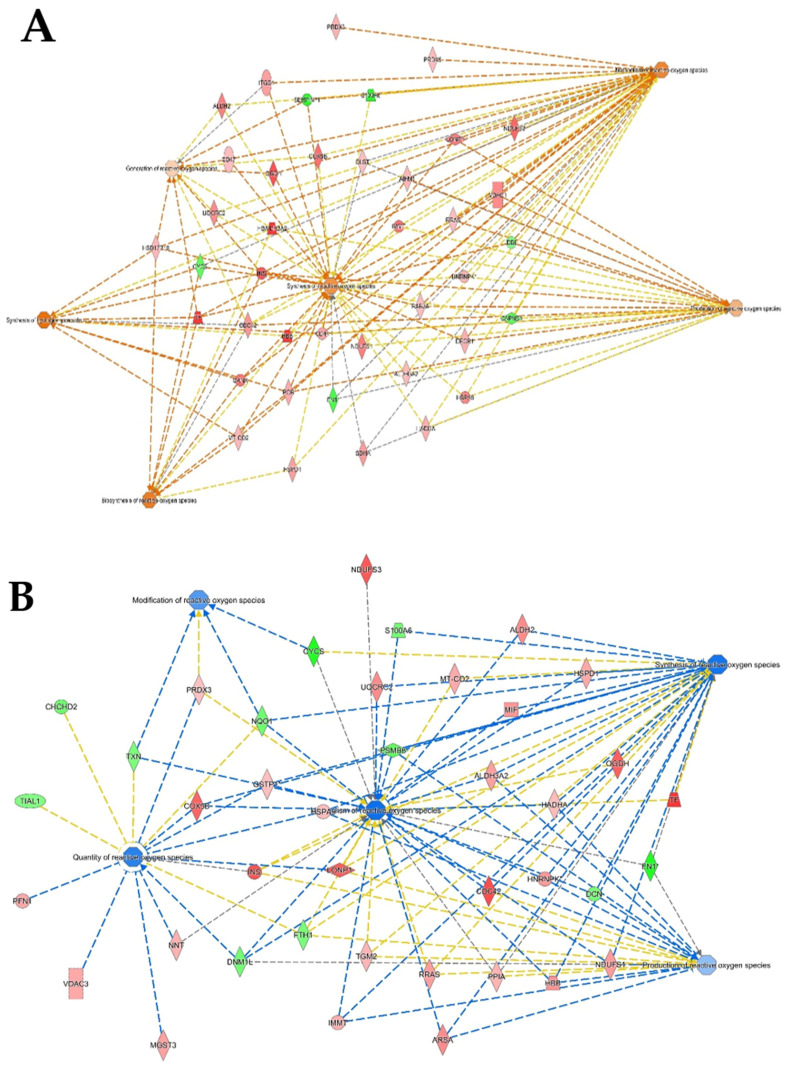
Functional modules related to pathways of reactive oxygen species obtained by IPA. (**A**) dermis spheroids at 14 days related to those at 7 days. (**B**) Carnosine treated spheroids at 7 days versus controls at 7 days. The orange and blue color of the central hub indicate an up and downregulation of the module, respectively, while red and green indicate the up and downregulated proteins, respectively.

To characterize the phenotype during growth and differentiation, human dermis spheroids collected at 14 days were correlated with ones collected at 7 days of culture by label free quantitative proteomics, based on high-resolution mass spectrometry. A total of 2171 proteins were quantified and 315 proteins were differentially regulated, of which 143 over and 42 under expressed, with a fold change higher than 1.5, at 14 related to 7 days of culture (Figure 2A, Supplementary Table S1). Several protein resulted regulated analyzing the differences between the group of 14 versus those of 7 days culture by a two-sided t-test. The high number of identified proteins allowed the description of all cellular compartments. Gene ontology terms of cellular components resulted in a wide distribution of identified proteins in the space of all the spheroids: 60% of proteins were cytoplasmic, 6% were in the extracellular space and 22% were nuclear followed by 9% belonging to plasma membrane (Figure 3A). Network analyses, based on differentially regulated proteins, showed a positive induction of oxidative phosphorylation (25 genes, z-score = 4.6, *p* value = 2.5 × 10^−26^) and mitochondrial activity (37 genes, *p* value = 9.9 × 10^−38^). Proteins such as NADH: ubiquinone oxidoreductase core subunit V2 (NDUFV2, log2 ratio = 1.59), cytochrome C oxidase subunit 5B (COX5B, log2 ratio = 1.27) and ATP synthase peripheral stalk subunit d (ATP5H, log2 ratio = 1.47) were over expressed, demonstrating the proper functionality of mitochondria. The production of ATP by energy derived from the electron transport system and the oxidation-reduction reactions in the mitochondria were positively induced. In addition, the TCA (tricarboxylic acid cycle) pathway was also upregulated to generate energy and reduced power by the ATP generating process (9 genes, z-score = 3, *p* value = 1.78 × 10^−12^). All the proteins correlated to this pathway, such as malate dehydrogenase 2 (MDH2, log2 ratio = 0.58), oxoglutarate dehydrogenase (OGDH, log2 ratio = 1.55) and citrate synthase (CS, log2 ratio = 0.81), were upregulated according to the expected behavior of high functionality of the TCA cycle. Forty-eight proteins, such as transferrin (TF, log2 ratio =3.98), vimentin (VIM, log2 ratio = 3.17), tubulin beta 4A class Iva (TBB4, log2 ratio = 1.94) and insulin (INS, log2 ratio = 1.59), belonging to the organization of the cytoplasm pathway had a measurement direction consistent with increased functionality (z-score = 2.07, *p* value = 6.67 × 10^−5^). Apoptosis signaling was downregulated (7 genes, z-score = −1.13, *p* value = 1.28 × 10^−4^). Quantitative proteomics highlighted 322 proteins, belonging to ECM, of which 124 were of extracellular space while 198 from plasma–membrane during the long-term cell culture. The complex cell-matrix interaction process was thoroughly described during dermis restoring and tissue remodeling by Reactome (Figure 4). In detail, 17 proteins of extracellular space, such as fibrillin 1 (log2 ratio = 2.01) and microfibril associated protein 2 (log2 ratio = 1.37), and 24 proteins of plasma membrane, such as trophoblast glycoprotein (log2 ratio = 1.20), were found to be differentially expressed (Table 1), showing a structured organization of spheroids during cell culture. All these proteins are involved in several ECM protein pathways, such as matrix organization, collagen homeostasis and elastic fiber formation (Figure 3B). Elastic and collagen fibers are components of the dermal ECM involved in the control of cutaneous elasticity [10]. In particular, collagen constitutes up to 30% of total protein in the ECM of multicellular animals [11]. It associates with elastic fibers, composed of elastin and fibrillin microfibrils, which give tissues the ability to recover after stretching. In this work, collagen type VI alpha 2 (COL6A2, log2 ratio = 0.85) and 3 chain (COL6A3, log2 ratio = 4.7) were upregulated. COL6 forms distinct microfibrils called beaded filaments, characterized by highly cross-linked disulphide branched network interwoven with fibrillar collagens [12]. It acts as an early sensor of the injury/repair response and may regulate fibrogenesis by modulating cell–cell interactions, stimulate the proliferation of mesenchymal cells and preventing cell apoptosis [13,14,15]. Elastin microfibril interface-located proteins (EMILIN-1 and 2, log2 ratio = 0.72 and 1.33, respectively), modulator of cell behavior, with growth factor and ECM assembly activity in the skin [16], were found here to be upregulated. Fibrillin-1, a large (∼350 kDa) cysteine-rich glycoprotein and the major structural component of microfibrils [17] was also found to be upregulated (log2 ratio = 2.00). Moreover, network analyses highlighted functional modules related to reactive oxidative species (ROS) involved during culture of the spheroids. The main free radical scavenging modules, such as ROS metabolism (z-score = 1.28, *p* value = 1.22 × 10^−15^), synthesis (z-score = 1.11, *p* value = 2.95 × 10^−14^) and production (z-score = 0.69, *p* value = 4.68 × 10^−8^) were over expressed (Figure 5A), indicating an increase of oxidative stress due to the physiological senescence of fibroblast during culture time.

### 2.2. Differential Regulation of Protein Pathways by L-Carnosine in Dermis Spheroids

Pharmacological treatment with carnosine prevents several diseases, such as metabolic syndrome and cardiovascular disorders [2]. Topical treatment of carnosine also has an anti-aging effect on skin, preventing UVA damage [6]. To describe the molecular action of carnosine on dermis, the previously described fibroblast spheroids model was treated with 150 µM carnosine. During the long-term culture the dermis phenotype showed an increase of oxidative stress related to senescence. For this reason, the effect of carnosine was studied at the intermediate time of culture and the treatment was stopped after 7 days. The two groups represented by the spheroids treated by carnosine at 7 days and the controls at 7 days showed significant differences. A total of 485 proteins were regulated, of which 115 up and 89 down expressed with a fold change higher than 1.5 (Figure 2B, Supplementary Table S1). The proteins belonging to ECM indicated a proper reorganization of matrix due to the contribution of plasma membrane proteins and extracellular space proteins (Table 1). In addition, proteins such as basigin (log2 ratio = 0.69), thymosin beta 4 X-linked (log2 ratio = 0.72) and tubulin beta-3 chain (log2 ratio = 0.73), showed a higher regulation operated by treatment of carnosine on spheroids (Supplementary Table S1). Basigin, a glycoprotein of membrane and a member of the immunoglobulin family, interacts with integrins and metalloproteases participating in the physiological and pathological ECM reorganization and cellular proliferation [18,19]. In this study, its activation highlights the role of carnosine in influencing the synthesis and proteolysis of matrix components during cellular differentiation. Thymosin beta 4 X-linked (TMSB4X) also plays a role in actin-based processes, such as cell migration and ECM remodeling [20,21]. TMSB4X also participates in wound healing of the epidermis interacting with actin cytoskeleton [22]. Its upregulation, due to carnosine treatment, further shows the role of carnosine in the maintenance of ECM processes (Figure 4, Table 1). Carnosine activated the functionality of mitochondria as demonstrated by protein network analyses. Functional modules, such as oxidative phosphorylation (23 genes, z-score = 4.38, *p* value = 4.73 × 10^−23^), TCA cycle (6 genes, z-score = 1.63, *p* value = 1.97 × 10^−7^) and ATP synthesis (9 genes, z-score = 2.11, *p* value = 2.85 × 10^−13^) were upregulated. The mitochondrial redox balance was also increased by carnosine as shown by the upregulation of cytochrome c oxidase subunit (5BCOX5B, log2 ratio = 1.41), a multi-subunit protein complex involved in the last step of the mitochondrial electron transport chain, and nicotinamide nucleotide trans-hydrogenase (NNT, log2 ratio = 0.7), an enzymatic regulator of mitochondrial NADPH (Supplementary Table S1 and Figure S1). In addition, the apoptosis pathway (5 genes, z-score = −2, *p* value = 6.03 × 10^−3^) was downregulated. Interestingly, carnosine induced a downregulation of functional modules related to ROS activities, such as their generation (11 genes, z-score = −1.66, *p* value = 3.46 × 10^−4^), metabolism (35 genes, z-score = −1.95, *p* value = 1.13 × 10^−11^), modification (4 genes, z-score = −1.00, *p* value = 4.97 × 10^−5^), production (19 genes, z-score = −0.58, *p* value = 1.88 × 10^−5^), synthesis (31 genes, z-score = −1.76, *p* value = 8.69 × 10^−10^) and amount (16 genes, z-score = −1.39, *p* value = 1.93 × 10^−7^) in dermis spheroids at 7 days of treatment related to proper control (Figure 5B). These pathways are tightly regulated by several proteins implicated in redox mechanisms. The protection from oxidative damage is operated by the activation of antioxidant detoxification genes. An example is microsomal glutathione S-transferase 3 (MGST3), which catalyzes the conjugation of reduced glutathione to protein targets in vitro [23]. Carnosine activated MGST3 (log2 ratio = 0.84) and GSTP1 (log2 ratio = 0.51), is an enzyme involved in the formation of glutathione conjugate with prostaglandins [24] at 7 days of treatment. Thioredoxin-related transmembrane protein 1 (TMX1, log2 ratio = 1.76), which catalyzed dithiol-disulfide exchange reaction, was also highly upregulated. Moreover, carnosine treatment on 3D dermis spheroids at 7 days of culture upregulated macrophage migration inhibitory factor (MIF, log2 ratio = 0.94), a multifunctional protein involved in oxidoreductase activity. MIF is able to reduce damage in the hearts of mice caused by oxidative stress and its deficiency induces an accumulation of ROS and senescence [25]. All this evidence attested and validated the role of carnosine in the protection against oxidative stress and aging.

## 3. Materials and Methods

### 3.1. Cell Source and Culture

Primary dermal fibroblasts isolated from a 50-year-old donor purchased from Innoprot (Derio, Spain), were cultured and amplified at early passages in CnT-PR F serum-free medium (CELLnTEC, Bern, Switzerland) in order to obtain 5 × 10^6^ cells.

### 3.2. Dermis Spheroid Production and L-Carnosine Treatment

Primary human dermal fibroblasts were detached once 90% of confluence was reached. Cells were washed twice with DPBS 1X (Merck Life Science S.r.l., Milan, Italy) and incubated with T/E solution (Primary Detach Kit, Innoprot, Derio, Spain) for 5 min. Cells were counted, centrifuged and seeded in Akura^®^Pate (InSphero AG, Schlieren, Switzerland) by ViaFlo Assist Plus (Integra Bioscience AG, Zizers, Switzerland) in order to obtain 10,000 cells/spheroids. Thanks to hanging drop technology, specific geometrical guidance allows cells to self-assemble and to aggregate forming round-shaped tridimensional scaffold-free spheroids. After 3 days in the hanging drop culture, spheroids were well formed and ready to be transferred into a GravityTrap^®^ plate (InSphero AG, Schlieren, Switzerland). Tissues were treated with 150 μM L-carnosine the day after, added to the culture medium and tissues were collected after 7 days of treatment. Fresh treatment was performed every other day. Three biological replicates of pooled 20 spheroids were collected, washed twice with cold DPBS 1X and allowed to settle to the bottom of conical tubes. After sedimentation, DPBS was discarded, and tissues were stored at −80 °C until the analysis.

### 3.3. Immunofluorescence for Collagen Type III on 3D Whole Mount Samples and Signal Quantification

Scaffold-free dermis spheroids cultured for 7 days (D7) and for 14 days (D14) untreated and treated with carnosine were collected for histological analysis. Tissues were washed in PBS 1X and fixed in formalin buffered solution 10% (Merck Life Science S.r.l., Milan, Italy) for 1 h at RT. After tissue fixation, samples were prepared for histological clearing before incubation with the primary antibody. Visikol Histo-M Starter Kit^®^ (Visikol, Hampton, NJ, USA) was used as a clearing reagent and samples were treated according to internal procedure (2): pooled samples (*n* = 4 DERMIS-spheroids/each pool) were dehydrated and rehydrated with cycles of 15 min each by incubations with increasing concentrations of ET-OH solutions (from 50% to 100%). After permeabilization with PBS-Triton solution 0.2% and Visikol Histo^®^ Permeabilization Buffer (Visikol, Hampton, NJ, USA), the blocking of specific sites was performed by incubation with Visikol Histo^®^ Blocking Solution (Visikol, Hampton, NJ, USA). Samples were incubated with collagen type III rabbit polyclonal primary antibody diluted in Visikol Histo^®^ Antibody Buffer (Visikol, Hampton, NJ, USA) overnight at 4 °C. Alexa 555 donkey anti-rabbit was used as a secondary antibody while nuclei were stained with DAPI (Merck, 1:2500). Stained dermis spheroids were cleared with Visikol Histo^®^-M overnight at 4 °C. The acquisitions were performed by Leica THUNDER DMi8 3D Cell Imager and Z-stacks video was acquired with a Leica sCMOS Camera and post-processed by LASX 3.0.1 software (Leica Microsystems Srl, Milan, Italy). Z-stacks were acquired in order to observe the whole volumes of 3D spheroids. Signal expression acquisition was optimized by the Thunder Computational Clearing algorithm and some more informative frames were extrapolated from the Z-stack video. The collagen type III expression signal was quantified by Leica LASX software (Leica Microsystems Srl, Milan, Italy).

### 3.4. Protein Extraction and in-Solution Trypsin Digestion

The cell pellets were resuspended in the solubilization buffer (8 M urea in 50 mM Tris–HCl, 30 mM NaCl, pH 8.5 and 1% protease inhibitor) and incubated on ice for 5–10 min. Cell lysates were further homogenized by sonication in an ice bath three times each for 15 s with 1 min intervals, using an ultra sonicator. Samples were centrifuged at 14,000 rpm for 20 min at 4 °C. The protein supernatant was collected into the new tube and pelleted cell debris was discarded. Samples were stored at −80 °C until they would be used for further experiments. The protein estimation was carried out by using the BCA assay; 10 µg of proteins was diluted in 50 mM NH_3_HCO_4_; and then reduced with 5 mM DL-dithiothreitol (DTT, Merck Life Science S.r.l., Milan, Italy) for 30 min at 52 °C, then centrifuged at 500 rpm and alkylated with 15 mM iodoacetamide (Merck Life Science S.r.l., Milan, Italy) for 20 min in the dark at room temperature. The trypsin digestion was performed in 1:20 enzyme/protein ratio (*w*/*w*) (Trypsin Sequencing Grade; Roche, Monza, Italy) overnight at 37 °C. The obtained peptides were desalted using zip-tip C18, then dried and stored at −20 °C before the analysis.

### 3.5. High-Resolution LC-MS/MS Analysis and Data Elaboration

Tryptic peptides were analyzed using a Dionex Ultimate 3000 nano-LC system (Sunnyvale, CA, USA) connected to an Orbitrap Fusion Tribrid Mass Spectrometer (Thermo Scientific, Bremen, Germany) equipped with a nano-electrospray ion source. Peptide mixtures were pre-concentrated onto an Acclaim PepMap 100—100 µm × 2 cm C18 and separated on EASY-Spray column, 15 cm × 75 µm ID packed with Thermo Scientific Acclaim PepMap RSLC C18, 3 µm, 100 Å. The chromatographic separation was performed at 35 °C and the flow rate was 300 nL/min. Mobile phases were as follows: 0.1% formic acid (FA) in water (solvent A); 0.1% FA in water/acetonitrile (solvent B) with a 2/8 ratio. Peptides were pre-concentrated at 96% of solvent A for 3 min and eluted from the column with the following gradient: 4% to 28% of B for 90 min and then 28% to 40% of B in 10 min, and to 95% within the following 6 min to rinse the column. The column was re-equilibrated for 20 min. Total run time was 130 min. One blank was run between triplicates to prevent sample carryover. MS spectra were collected over an m/z range of 375–1500 at 120,000 resolutions, operating in the data dependent mode with a cycle time of 3 s. Higher-energy collisional dissociation (HCD) was performed with collision energy set at 35%. Each sample was analyzed in three technical triplicates. Resulting MS raw data from all the technical and biological replicates were analyzed by using MaxQuant software (version 1.6.2.3, Max Planck Institute of Biochemistry, Martinsried, Germany). The Andromeda search engine was used to identify MS/MS based peptide and proteins in MaxQuant using a target-decoy approach with less than 1% of false discovery rate (FDR). In the present study we used the *Homosapiens* database (https://www.uniprot.org/proteomes/UP000005640, accessed on 24 January 2022). Trypsin was selected as a cutting enzyme; two missed cleavages and a maximum of five modifications per peptide were allowed. Methionine oxidation and acetylation (N terminus) was used as a variable modification. Carbamidomethylation was used as a fixed modification. The proteins were selected with a minimum of two peptides. For the label-free quantification of proteins, we applied the MaxLFQ algorithm (version 1.6.2.3, Max Planck Institute of Biochemistry, Martinsried, Germany). Matching between the runs option was enabled and the remaining default parameters were permitted. An open-source Perseus software (version 1.6.1.3; Max Planck Institute of Biochemistry, Martinsried, Germany) was used for the identification of statistically significant and differentially regulated proteins. The interpretation and visualization of results obtained from the MaxQuant software was performed by a two-sample t-test using Perseus (v1.6.1.3, Max Planck Institute of Biochemistry, Martinsried, Germany). Statistical parameters (*p* < 0.05; q < 0.05, q = FDR adjusted *p* value) were set to identify the differentially expressed proteins between samples. Variabilities of biological replicates were measured with Pearson correlation coefficient values of the LFQ intensities. The differentially regulated proteins had a minimum of two peptides and FDR adjusted *p* value.

### 3.6. Protein Network Analysis

The protein network analysis, related to significantly modulated proteins, was carried out by the ingenuity pathways analysis (IPA) (QIAGEN Aarhus Prismet, Denmark, September 2021). The statistical enrichment of the involved pathways was performed by the right-tailed Fisher’s exact test, in correlation with the QIAGEN Knowledge Base, assigning a *p* value. The core analyses performed by IPA, using the differentially expressed proteins in the uploaded dataset, assess signaling pathways, molecular interaction network and biological functions that can likely be perturbed. The overall activation/inhibition states of canonical pathways are predicted through a z-score algorithm. This z-score is used to statistically compare the uploaded dataset with the pathway patterns. The pathways are colored to indicate their activation z-scores: orange predicts a gain of function, while blue a loss of function. The pathway is activated when molecules’ causal relationships with each other (i.e., activation edge and the inhibition edge between the molecules based on literature findings) generate an activity pattern for the molecules and the end-point functions in the pathway. Reactome (https://reactome.org, accessed on 1 July 2021) pathway analysis was performed for evaluating ECM cell-matrix interaction [26].

## 4. Conclusions

We found that 3D micro physiological models of human tissues and quantitative proteomics provide a useful advanced tool for in depth tissue characterization. The development of an innovative tissue platform, based on a miniaturized model close to the natural tissue phenotype and the evaluation of protein crosstalk by active modules of defined signaling and pathways, pave the way to a new more complete approach suitable for personalized care therapies. The development of a donor-like tissue model and the functional sequencing of modulated proteomic profiles allowed the in-depth characterization of cell compartment assembly, mitochondrial processes, cytoskeleton reorganization and apoptosis. The cellular pathway description in a healthy state is the starting point to analyze the proteome variation of the biological system under stress or under drug treatment. The variation of the protein profile related to the healthy state allows for understanding of the molecular mechanism involved in stress or induced by drugs by the identification and quantification of proteins and their modulated pathways. In the present study, the integrated approach of network analyses based on high-resolution mass spectrometry showed the physiological phenotype of spheroids and the ECM mirroring of the native in vivo dermis features. The response to carnosine treatment highlighted the modulation of important pathways related to the ECM reorganization, apoptosis and free radical scavenging. In conclusion, the innovative tissue system of micro-physiological dermal spheroids and the advanced quantitative proteomics approach demonstrated the key role of carnosine in the protection of dermis compartment against the physiological oxidative stress and natural aging, which occurs over time.

## Figures and Tables

**Table 1:** Extracellular matrix proteins in spheroids at 14 days versus ones at 7 days (CD14 vs. CD7) and carnosine treated spheroids at 7 days versus control spheroids (PD7 vs. CD7).

Gene Name	Protein Name	*p* Value C7 vs. C14	Log_2_ C7/C14	*p* Value PD7 vs. C7	Log_2_ PD7/C7	Location
TF	transferrin	1.35 × 10^−07^	3.98	8.78 × 10^−06^	2.67	Extracellular Space
COL6A3	collagen type VI alpha 3 chain	1.16 × 10^−03^	4.71	2.64 × 10^−03^	2.28	Extracellular Space
STOML2	stomatin like 2	2.77 × 10^−02^	1.36	5.00 × 10^−05^	2.13	Plasma Membrane
FBN1	fibrillin 1	3.61 × 10^−02^	2.01	1.11 × 10^−01^	1.93	Extracellular Space
INS	insulin	1.42 × 10^−02^	1.53	4.84 × 10^−04^	1.56	Extracellular Space
SLC1A5	solute carrier family 1 member 5	7.57 × 10^−04^	1.54	7.41 × 10^−04^	1.46	Plasma Membrane
TPBG	trophoblast glycoprotein	2.28 × 10^−03^	1.20	5.37 × 10^−04^	1.45	Plasma Membrane
MFAP2	microfibril associated protein 2	1.31 × 10^−02^	1.37	8.77 × 10^−03^	1.42	Extracellular Space
GPNMB	glycoprotein nmb	1.88 × 10^−02^	1.17	1.45 × 10^−02^	1.36	Plasma Membrane
MIF	macrophage migration inhibitory factor	2.26 × 10^−02^	−0.49	5.21 × 10^−03^	0.94	Extracellular Space
RAP2B	RAP2B. member of RAS oncogene family	7.48 × 10^−03^	0.94	3.60 × 10^−03^	0.93	Plasma Membrane
MME	membrane metalloendopeptidase	5.19 × 10^−02^	0.76	5.59 × 10^−02^	0.89	Plasma Membrane
CCDC47	coiled-coil domain containing 47	7.94 × 10^−02^	0.91	4.60 × 10^−02^	0.72	Extracellular Space
LAMTOR1	late endosomal/lysosomal adaptor. MAPK and MTOR activator 1	1.78 × 10^−02^	1.31	1.46 × 10^−01^	0.70	Plasma Membrane
BSG	basigin (Ok blood group)	8.96 × 10^−01^	0.03	5.41 × 10^−04^	0.69	Plasma Membrane
EMILIN2	elastin microfibril interfacer 2	2.38 × 10^−03^	1.33	2.57 × 10^−02^	0.69	Extracellular Space
MANF	mesencephalic astrocyte derived neurotrophic factor	8.26 × 10^−02^	0.32	7.10 × 10^−03^	0.68	Extracellular Space
ITGAV	integrin subunit alpha V	6.55 × 10^−03^	0.50	2.10 × 10^−02^	0.63	Plasma Membrane
LOXL1	lysyl oxidase like 1	8.17 × 10^−01^	0.05	2.39 × 10^−03^	0.62	Extracellular Space
CD248	CD248 molecule	3.71 × 10^−03^	0.81	6.14 × 10^−02^	0.62	Plasma Membrane
SCARB2	scavenger receptor class B member 2	3.63 × 10^−03^	1.10	1.65 × 10^−01^	0.60	Plasma Membrane
NT5E	5’-nucleotidase ecto	3.15 × 10^−05^	1.10	9.54 × 10^−02^	0.59	Plasma Membrane
ARL8B	ADP ribosylation factor like GTPase 8B	9.04 × 10^−02^	0.66	1.07 × 10^−01^	0.57	Plasma Membrane
THY1	Thy-1 cell surface antigen	4.21 × 10^−04^	1.30	1.61 × 10^−01^	0.55	Plasma Membrane
COL6A2	collagen type VI alpha 2 chain	1.60 × 10^−02^	0.85	1.22 × 10^−01^	0.52	Extracellular Space
LAMP1	lysosomal associated membrane protein 1	2.76 × 10^−01^	0.36	5.61 × 10^−02^	0.52	Plasma Membrane
ESYT2	extended synaptotagmin 2	2.21 × 10^−02^	0.66	1.22 × 10^−01^	0.50	Plasma Membrane
CD81	CD81 molecule	4.55 × 10^−03^	0.88	1.43 × 10^−01^	0.46	Plasma Membrane
CD44	CD44 molecule (Indian blood group)	1.80 × 10^−02^	0.82	2.69 × 10^−01^	0.44	Plasma Membrane
EMILIN1	elastin microfibril interfacer 1	1.91 × 10^−02^	0.72	5.22 × 10^−02^	0.36	Extracellular Space
HLA-A	major histocompatibility complex. class I. A	3.99 × 10^−03^	0.89	2.65 × 10^−01^	0.35	Plasma Membrane
MARCKS	myristoylated alanine rich protein kinase C substrate	9.03 × 10^−02^	−0.26	9.51 × 10^−04^	−0.77	Plasma Membrane
LAMP2	lysosomal associated membrane protein 2	7.14 × 10^−01^	0.07	1.63 × 10^−02^	−0.77	Plasma Membrane
PLPP3	phospholipid phosphatase 3	8.12 × 10^−01^	0.03	2.23 × 10^−02^	−0.85	Plasma Membrane
FN1	fibronectin 1	2.20 × 10^−09^	−0.84	2.50 × 10^−01^	−1.46	Extracellular Space

## Data Availability

The data presented in this study are available in Supplementary Materials and in PRIDE repository (PXD031053).

## Supplementary Materials

The following supporting information can be downloaded at: https://www.mdpi.com/article/10.3390/ijms23031468/s1.
