# Supplementary material for: Oxidative Stress Modulation by Carnosine in Scaffold Free Human Dermis Spheroids Model: A Proteomic Study
Source: Int J Mol Sci. 2022 Jan 27;23(3):1468. doi: 10.3390/ijms23031468 (PMC8836079; doi:10.3390/ijms23031468)
Supplement: Supplementary file 1 [file ijms-23-01468-s001.zip › manuscript.v7revised/SupplementaryFigure1_2.pptx]

## Slide 1
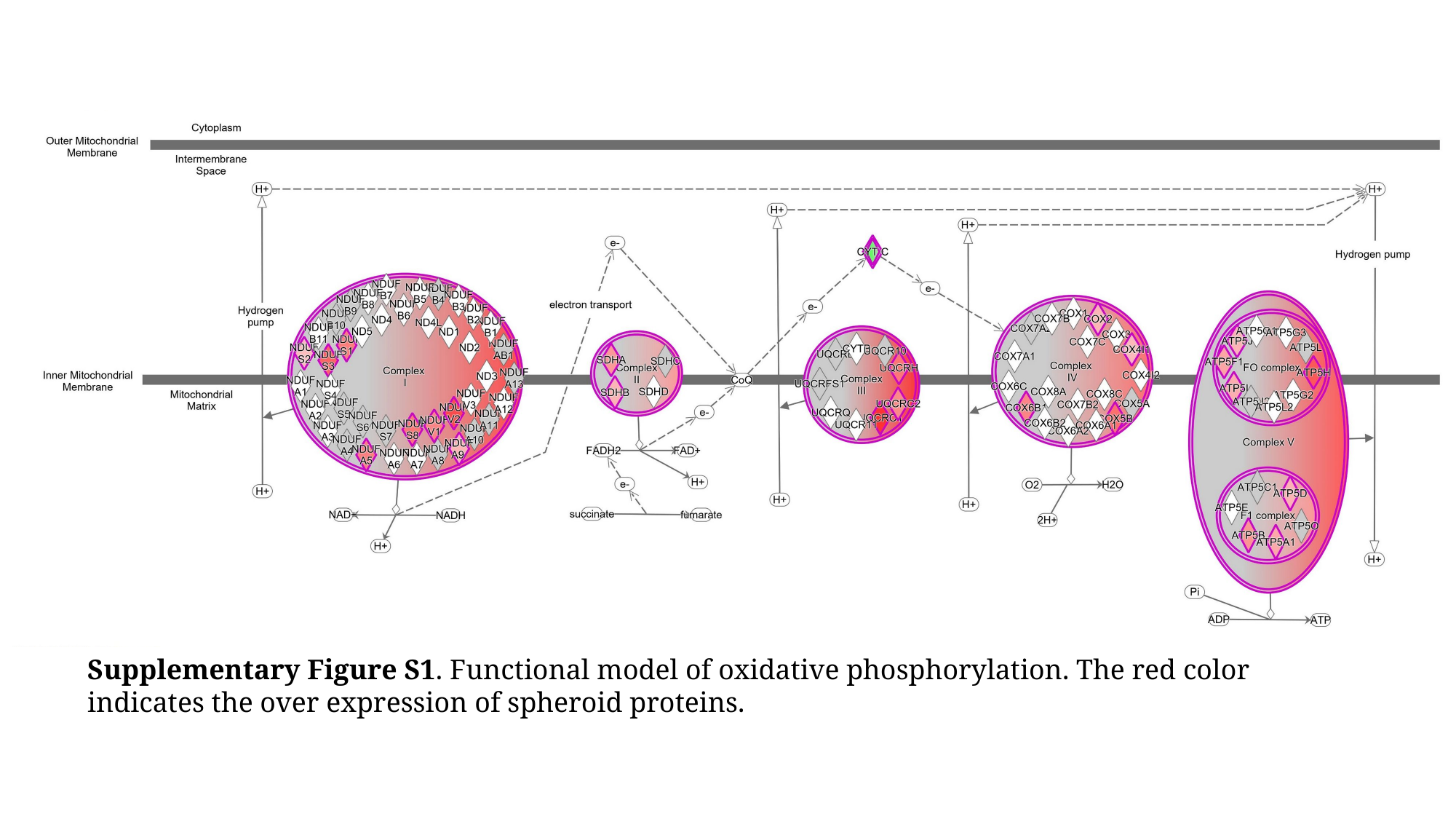

Supplementary Figure S1. Functional model of oxidative phosphorylation. The red color indicates the over expression of spheroid proteins.

## Slide 2
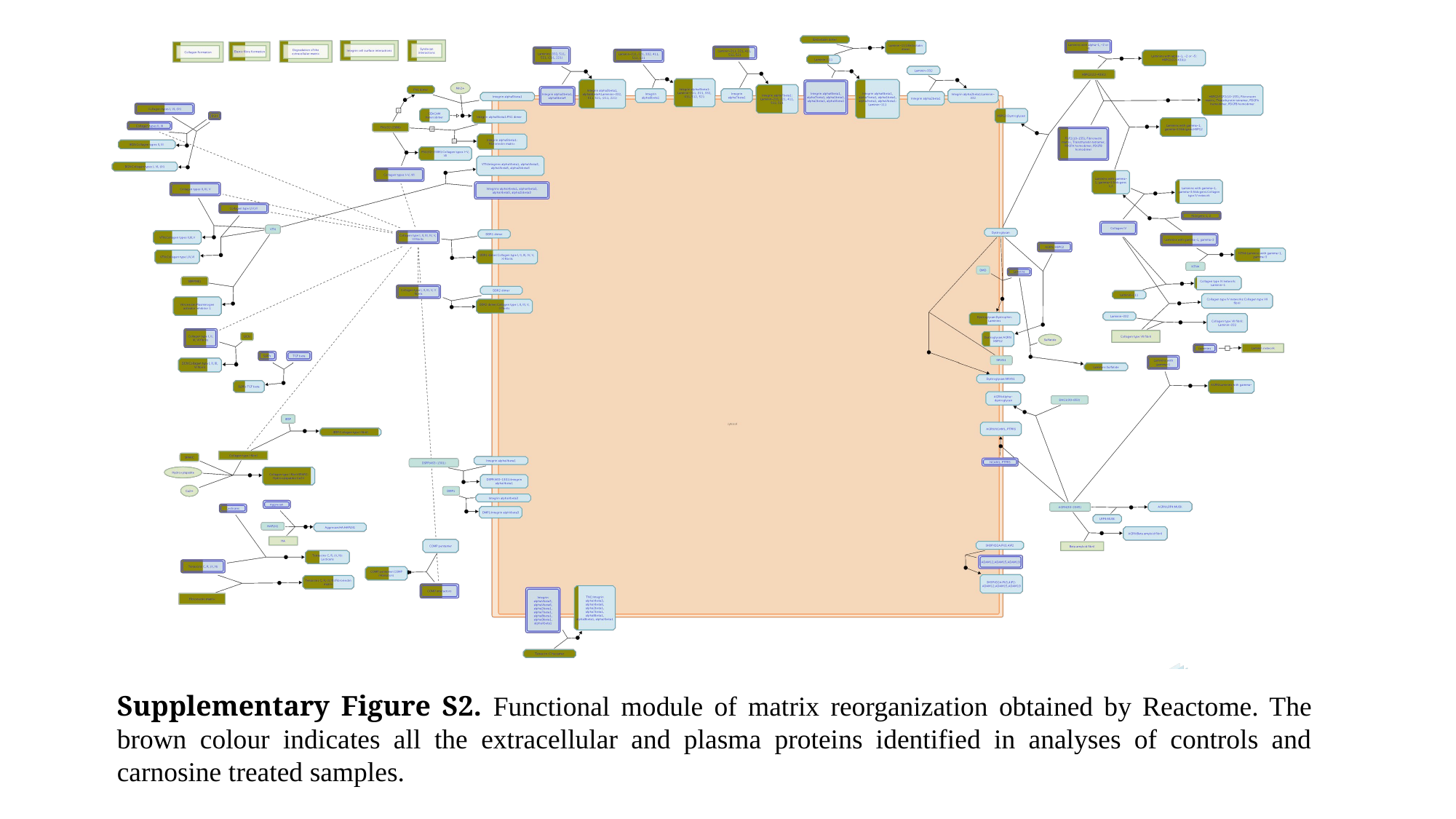

Supplementary Figure S2. Functional module of matrix reorganization obtained by Reactome. The brown colour indicates all the extracellular and plasma proteins identified in analyses of controls and carnosine treated samples.
